# Effect of Crosslinking Type on the Physical-Chemical Properties and Biocompatibility of Chitosan-Based Electrospun Membranes

**DOI:** 10.3390/polym13050831

**Published:** 2021-03-09

**Authors:** Andrea Dodero, Sonia Scarfi, Serena Mirata, Alina Sionkowska, Silvia Vicini, Marina Alloisio, Maila Castellano

**Affiliations:** 1 Department of Chemistry and Industrial Chemistry, University of Genoa, Via Dodecaneso 31, 16146 Genoa, Italy; marina.alloisio@unige.it (M.A.); maila.castellano@unige.it (M.C.); 2 Department of Earth, Environment and Life Sciences (DISTAV), University of Genoa, Via Pastore 3, 16132 Genoa, Italy; sonia.scarfi@unige.it (S.S.); serena.mirata@edu.unige.it (S.M.); 3 Inter-University Center for the Promotion of the 3Rs Principles in Teaching & Research (Centro 3R), Via Caruso 16, 56122 Pisa, Italy; 4 Department of Chemistry of Biomaterials and Cosmetics, Nicolaus Copernicus University in Toruń, Gagarin 7, 87-100 Toruń, Poland; alinas@umk.pl

**Keywords:** chitosan, electrospun nanofibers, crosslinking, physical-chemical properties, biocompatibility

## Abstract

Chitosan nanofibrous membranes are prepared via an electrospinning technique and explored as potential wound healing patches. In particular, the effect of a physical or chemical crosslinking treatment on the mat morphological, mechanical, water-related, and biological properties is deeply evaluated. The use of phosphate ions (i.e., physical crosslinking) allows us to obtain smooth and highly homogenous nanofibers with an average size of 190 nm, whereas the use of ethylene glycol diglycidyl ether (i.e., chemical crosslinking) leads to rougher, partially coalesced, and bigger nanofibers with an average dimension of 270 nm. Additionally, the physically crosslinked mats show enhanced mechanical performances, as well as greater water vapour permeability and hydrophilicity, with respect to the chemically crosslinked ones. Above all, cell adhesion and cytotoxicity experiments demonstrate that the use of phosphate ions as crosslinkers significantly improves the capability of chitosan mats to promote cell viability owing to their higher biocompatibility. Moreover, tuneable drug delivery properties are achieved for the physically crosslinked mats by a simple post-processing impregnation methodology, thereby indicating the possibility to enrich the prepared membranes with unique features. The results prove that the proposed approach may lead to the preparation of cheap, biocompatible, and efficient chitosan-based nanofibers for biomedical and pharmaceutical applications.

## 1. Introduction

**Figure 1:**
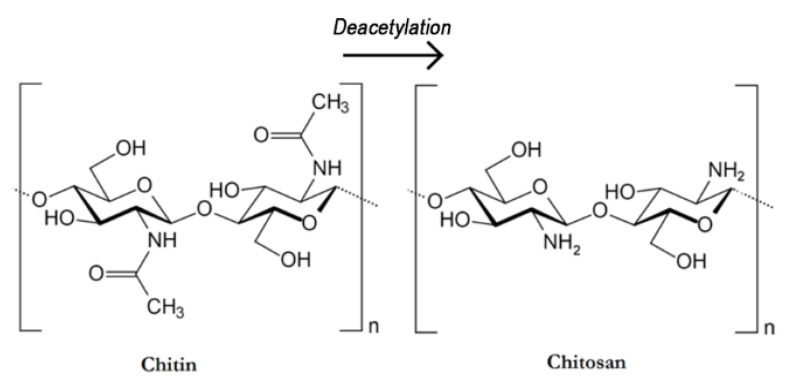
Chemical Structure of Chitin and Chitosan.

Chronic and traumatic wounds represent one of the biggest threats to human quality of life due to their high incidence and because they require long, expensive and, to date, poorly satisfactory medical treatments. Tissue reparation, which is a natural process, is often diminished by a broad number of factors with the employment of wound healing patches currently assuming a highly important role [1]. Nowadays, autologous, allogeneic, or xenogeneic grafting approaches are usually employed to the purpose despite the existence of several disadvantages ranging from the low availability to the possibility of immunogenic reactions [2,3]. On these bases, it is not surprising that the development of artificial systems able to induce tissue regeneration by promoting cell viability and, at the same time, providing a protective effect towards the external environments represents an important research field [4,5]. Despite the fact that, in previous years, several types of wound healing patches, such as films and three-dimensional hydrogels [6], have been prepared by using both synthetic and natural polymers [7], most of these products cannot completely satisfy the application requirements. Indeed, wound healing patches must possess a broad number of specific physical–chemical properties that should match those of human tissues along with marked biocompatibility [8,9], which are often difficult to obtain with the traditional fabrication approaches. In this regard, electrospinning [10] has emerged as a promising alternative technique for the preparation of nanofibrous scaffolds with a structure that strongly resembles the extra-cellular matrix (ECM), hence providing the ideal environment to foster cell viability [11,12,13]. Nanofibrous mats are indeed characterized by a high surface area and porosity, which makes them able to rapidly adsorb wound exudates and to allow gas exchange [14,15]. Additionally, nanoparticles of different natures [16,17], growth factors [18], drugs [19,20], and other bioactive compounds [21] can be easily embedded within the membranes leading to the preparation of functionalized nanofibers with unique properties. In addition, nearly any synthetic and natural polymer can be electrospun, the latter offering much greater advantages especially owing to their higher biocompatibility and biodegradability [22,23,24,25]. In this sense, chitosan (CS) is currently assuming a major role in substituting the frequently used synthetic polymers due to its unique properties [26]. Chitosan (Figure 1) is a cationic linear polysaccharide consisting of β-(1–4) linked d-glucose units and it is derived from the partial deacetylation of chitin, the second most abundant polymer in nature after cellulose [27]. The existence of amine groups on chitosan chains enables distinctive biological functions such as biocompatibility, biodegradability, bioactivity, non-toxicity, and good adsorption properties that make such polysaccharide extremely promising for biomedical and pharmaceutical purposes [28,29,30]. However, it should be kept in mind that the efficient electrospinning of chitosan and other naturally derived polymers still represents a challenge and some experimental attentions (i.e., use of synthetic co-spinning agents and surfactants) are required [31,32]. Additionally, despite the low water solubility of the raw material, chitosan-based nanofibers usually need to be subjected to coagulation and/or crosslinking treatments to increase their stability and integrity in aqueous environments [33]. In this sense, to date, the commonly employed approaches rely on the use of hazardous solvents and/or chemicals, which could, in turn, lead to cytotoxicity issues and reduce the applicability of chitosan-containing biomedical products [34,35].

**Figure 2:**
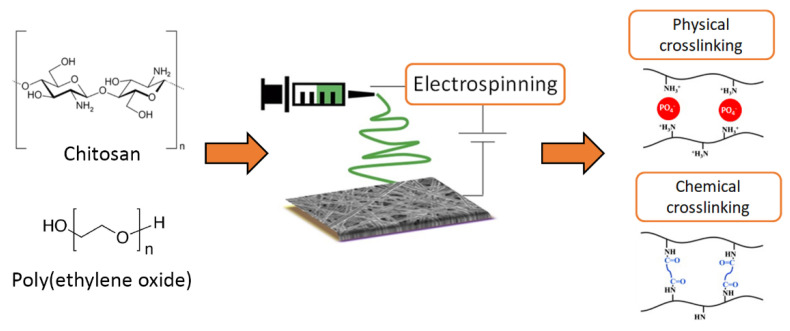
Schematic of the fabrication procedure of chitosan-based membranes.

With these premises, in a previous work, the electrospinning of chitosan solutions was optimized by using poly(ethylene oxide) as a co-spinning agent, which was subsequently completely removed, and a physical or a chemical crosslinking treatment was specifically applied to enhance the fiber stability [36]. Here, the proposed membranes are fully characterized in terms of their morphological, mechanical, water-related, and biological properties aiming to explore the possibility to exploit them as efficient wound healing patches. As such, the adsorption–desorption capability of the mats is as well investigated by using two model drug molecules. In this regard, the physical-crosslinked membranes were hypothesized to be more biocompatible with respect to those subjected to a chemical crosslinking reaction with the obtained results, proving their promising applicability in promoting tissue regeneration and/or reparation. Figure 2 summarizes the mat fabrication procedure.

## 2. Materials and Methods

### 2.1. Materials

Low molecular weight chitosan was obtained from Sigma-Aldrich and used without further purification. A degree of deacetylation (DD) of 78% was estimated via a conductometric titration method [37]. Poly(ethylene oxide) (PEO) with M_v_ = 900 kDa, Triton^TM^ X-100 laboratory-grade, glacial acetic acid, absolute ethanol (EtOH), ammonium hydroxide (NH4OH), sodium phosphate dibasic (Na2HPO4), and ethylene glycol diglycidyl ether (EGDE) (technical, ~50% (GC)), thiazolyl blue tetrazolium bromide (MTT), and dimethyl-sulfoxide (DMSO) were purchased from Sigma Aldrich (Milan, Italy). Saos-2 human osteoblast cell line and mouse fibroblast L929 cell line were obtained from the American Type Culture Collection (LGC Standards Srl, Milan, Italy). Human keratinocyte HaCaT cell line (CLS Cell Lines Service, 300493) was obtained by the Cell Lines Service (GmbH, Eppelheim, Germany). DMEM cell medium, glutamine, Fetal Bovine Serum (FBS), penicillin/streptomycin antibiotic solution and ready-to-use trypsin solution were purchased from Microstech Srl (Naples, Italy). Tissue culture flasks and 96-well plates were purchased from VWR International (Milan, Italy).

### 2.2. Methods

#### 2.2.1. Solution Preparation

CS-PEO batch solution to be electrospun was prepared as previously reported [36]. Firstly, CS powder was dissolved in a 5 *v*/*v*% acetic acid aqueous solution under gentle stirring for 24 h at room temperature. Subsequently, PEO powder was added and the mixture was kept under gentle stirring for further 24 h at room temperature. The polymer total concentration and ratio were 7 wt% and 1:1 (i.e., CS concentration of 3.5 wt% and PEO concentration of 3.5 wt%), respectively. Then, the system was added with 1 wt% of Triton and maintained under gentle stirring for 24 h at room temperature and finally conserved at T = 4 °C.

#### 2.2.2. Electrospinning and Membrane Crosslinking

Doxa Microfluidics^®^ (Malaga, Spain) Professional Electrospinning Machine equipped with an aluminium flat collector was employed to electrospin the prepared chitosan-based solution. A detailed description of the fabrication procedure optimization is reported in a previously published work [36]. In a typical experiment, 10 mL of solution were electrospun using a glass syringe connected to a 22 G flat-tip needle. A spinneret–collector distance of 20 cm, an infuse rate of 0.15 mL/h, and an applied voltage of 17.5 kV were used as working parameters. Temperature and humidity were controlled at T = 25 °C and 50% RH, respectively. Electrospun mats were carefully peeled off the aluminium collector and dipped for 30 min in a coagulation bath, which consisted of EtOH/NH_4_OH/H_2_O 7/2/1 at pH = 7.5, to neutralize the acetic acid and prevent the dissolution of chitosan in aqueous environments. The samples were then dried in an oven at T = 50 °C under vacuum overnight and subsequently immersed for 3 h in a crosslinking solution. Physically and chemically crosslinked membranes were prepared by using a 10 *w*/*v*% Na_2_HPO_4_ aqueous solution or a 2.5 *v*/*v*% EGDE aqueous solution heated up at T = 60 °C, respectively. Finally, chitosan-based mats were washed several times with deionized water and dried in an oven at T = 50 °C under vacuum overnight before being stored in a desiccator and characterized. It is noteworthy that chitosan chemical crosslinking was carried out at a temperature of 60 °C in order to accelerate the reaction hence reducing the loss of the nanofibrous structure.

#### 2.2.3. Morphological Investigation

Membrane morphology was evaluated via field-emission scanning electron microscopy (FESEM) through ZEISS (Oberkochen, Germany) SUPRA 40 VP operating at 10 kV in direct detector configuration (InLens). Good conductivity of the samples was assessed with a thin layer of sputter-coated carbon using a Polaron (Laughton, UK) E5100. ImageJ (National Institutes of Health, Bethesda, MD, USA) open-source software was employed to analyse the nanofiber dimension. At least 200 diameter measurements were taken for each of the high-magnification micrographs.

#### 2.2.4. Mechanical and Water-Related Characterization

Uniaxial tensile test was performed on the crosslinked mats by using a displacement-controlled dynamometer Instron 5565 (Instron, Turin, Italy). At least five rectangular specimens (40 × 10 mm) were tested for each sample. The thickness of the membranes was evaluated via a precision digital micrometer. A pre-load of 0.1 N and an elongation rate of 25 mm/min were employed for all the experiments. The Young modulus E, the tensile strength (σ_b_), and the elongation at break (ε_b_) were calculated from the obtained stress-deformation curves.

Water contact angle (WCA) of the crosslinked membranes was measured via an Attention Theta Lite optical tensiometer (Biolin Scientific, Gothenburg, Sweden). A small drop of water (i.e., volume = 3 μL) was placed on the sample surface and both right and left WCA were calculated via the instrument software.

Water vapour permeability (WVP) of the crosslinked membranes was assessed by means of a gravimetrical method according to ASTM E96-95. Briefly, circular samples were mounted on measuring cups with a diameter of 9.5 mm which were filled with distilled water up to 2 cm underneath the film. The cups were placed in an environmental chamber at 37 °C and 50% RH and weighted every hour for a period of 8 h. WVP was calculated as follows:

WVP = (WVTR·d)/(A·∆p)(1)

where WPVR is the water vapour transmission rate (g/s), d (m) is the thickness of the sample, A (m^2^) is the surface of the sample that permits the vapour diffusion, and ∆p (Pa) is the partial water vapour pressure difference across the two sides of the sample.

The moisture content (MC) of the crosslinked membranes was determined by placing a small piece of each sample at T = 110 °C under vacuum for 24 h and evaluating the weight loss after the drying process. MC percentage on a wet-basis was expressed as:

%MC = ((M_i_ − M_f_)/M_i_)·100 (2)

where M_i_ and M_f_ are the weights of the sample before and after the drying, respectively.

#### 2.2.5. Biological Tests

All cell lines were cultured at T = 37 °C in a humidified, 5% CO_2_ atmosphere in high glucose Dulbecco’s modified Eagle’s medium (DMEM), with glutamine supplemented wi th 10% FBS using penicillin/streptomycin as antibiotics. Experiments were performed in triplicate on 96-well plates. Crosslinked chitosan membranes were cut to obtain circular discs of 6 mm Ø and autoclaved before being positioned at the bottom of the wells.

To evaluate the eventual cytotoxicity, crosslinked chitosan mats were soaked in complete cell medium and incubated in the 96-well plates with 100 μL of medium per well for 6 h at T = 37 °C. After the incubation with the membranes, the cell medium from each well was collected and added to 96-well plates containing the cell cultures (10,000 cell/well in quadruplicate for each condition) with a final dilution of the extracts of 1:1. After 24 h, cell viability was measured by the MTT test as already described [38].

To evaluate cell adhesion on chitosan-based nanofibers, the cells were seeded at 10000 cells/well onto the membranes at T = 37 °C and incubated for 16 h. For each cell line, a standard curve was seeded in quadruplicate in the 96-wells in the 1250–10,000 cells/well range to obtain a linear regression curve to fit the experimental results and extrapolate the number of cells attached to the different membranes. At the end of the experiments, cell viability was assayed by MTT test as previously described [39]. Briefly, the mats were removed from the wells, washed one time by immersion in sterile phosphate buffer saline (PBS) and positioned in a new 96-well plate where a solution of 0.5 mg/mL MTT in complete medium was added and again incubated for 2 h at T = 37 °C in a humidified atmosphere. Subsequently, the medium was removed, 200 μL of DMSO was added to each well and allowed to dissolve the formazan salts onto the membranes for 5 min at room temperature. The membranes were then removed, and the absorbance of the wells was measured by a microplate reader at 540 nm.

Data are the means ± S.D. of three independent experiments.

#### 2.2.6. Adsorption–Desorption Properties

Adsorption and desorption properties of the physically crosslinked electrospun mats were investigated by using methylene blue (MB) and methyl orange (MO) as model drug molecules of opposite charge. UV-vis absorption spectra were acquired utilizing a UV-1800 spectrophotometer (Shimadzu, Kyoto, Japan) at room temperature.

In a typical kinetic adsorption study, 5 mg of sample were placed within a fused silica cuvette with 1 cm pathlength and soaked with 3.5 mL of dye aqueous solution at a given concentration (i.e., 5, 10 and 20 mg/L). The system was gently shaken for 6 h at room temperature and the solution dye concentration was periodically monitored.

In a typical isotherm adsorption experiment, 5 mg of the membrane were immersed in 50 mL of dye solutions with increasing concentration (i.e., 5, 10, 20, 40, 80, and 160 mg/L) under gentle shaking for a week at room temperature and then the supernatant dye concentration was analysed.

In a typical desorption experiment, a dye-loaded membrane was rapidly washed with EtOH and placed in a 1 cm pathlength fused silica cuvette. As the release medium, 3.5 mL of PBS at pH = 7.4 and T = 37 °C were used to simulate physiological conditions. The system was kept under gently stirring and the concentration of the supernatant was periodically monitored for 6 h.

The dye absorption capacity at a given time t (q_t_), at equilibrium (q_e_), and the cumulative release percentage (R%) were determined as:

q_t_ = ((C_0_ − C_t_)·V)/M (3)

q_e_ = ((C_0_ − C_e_)·V)/M (4)

R% = (m_r_/m_i_)·100 (5)

where C_0_, C_t_, C_e_ (mg/L) are the dye concentration at the initial time t_o_, at a given time t and equilibrium, respectively, V (L) is the solution volume, M (g) is the weight of the membrane, m_i_ (mg) is the dye load in the membrane at the time t_0_, and m_r_ (mg) is the dye amount released at time t.

## 3. Results and Discussion

### 3.1. Membrane Morphology

**Figure 3:**
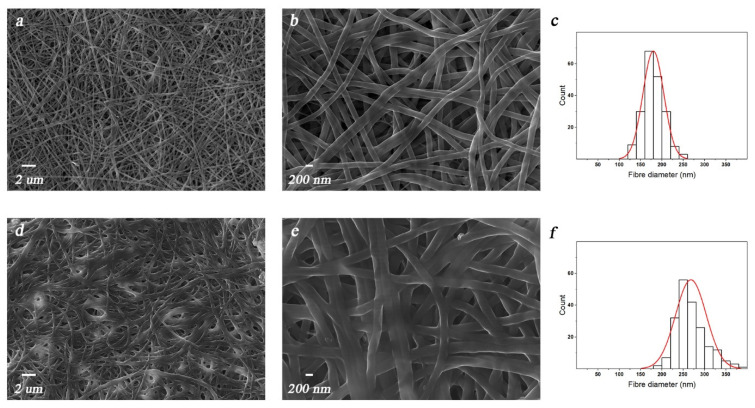
FESEM micrographs for chitosan membranes after (**a**,**b**) a physical or (**d**,**e**) a chemical crosslinking treatment and (**c**,**f**) their related size histograms.

Crosslinked electrospun chitosan-based membranes prepared by using PEO as co-spinning agent and poorly concentrated acetic acid aqueous solution as a solvent were recently reported [36]. More in detail, both a physical and chemical crosslinking approach were specifically developed and optimized by using phosphate ions and ethylene glycol diglycidyl ether, respectively, aiming to endow the nanofiber with high stability in physiological conditions. Remarkably, owing to its great water solubility, PEO was completely removed from the nanofibers during the crosslinking reaction as proved by thermogravimetric analysis and Fourier-transform infrared spectroscopy (data reported and discussed in [36]). The morphology of the mats after the physical or chemical crosslinking treatment is shown in Figure 3a,b,d,e, respectively, with the related size distribution histograms reported in Figure 3c,f.

Despite a well-defined and homogenous nanostructure being obtained independently on the crosslinking approach, significant morphological differences could be observed between the two prepared samples. More in detail, physically crosslinked mats were characterized by thinner (i.e., average diameter of 190 nm) and smoother nanofibers that completely maintained their individuality, thereby leading to the formation of a high number of interconnected pores. On the contrary, the chemical crosslinking treatment promoted the partial coalescence of the nanofibers (i.e., an average diameter of 270 nm), which in turn led to the establishment of a denser nanostructure presenting an average low porosity. Such findings are, to some extent, unexpected but may be most likely ascribable to the dissimilar reticulation mechanism and kinetic. Indeed, chemical crosslinking occurs via the formation of strong, stable, and not reversible covalent bonds between chitosan chains, whereas HPO_4_^−2^ ions can only act as weak and reversible links. However, such temporary crosslinking points require a much shorter time to be formed with respect to the covalent bonds, as they are rapidly created when chitosan comes into contact with the phosphate ions. In contrast, the chemical reaction occurring between chitosan amino groups and EGDE epoxy groups necessitates a certain time to happen, during which the nanofibers tend to swell, thereby increasing their size and partially collapsing and/or coalescing with each other [40]. In any case, despite of the described differences, both the proposed mats showed a suitable structure to foster cell viability owing to their great resemblance with the extra-cellular matrix [41,42].

### 3.2. Mechanical and Water-Related Properties

Mechanical and water-related properties play a fundamental role in most biomedical devices where the existence of applied stresses and the existence of interfaces with native tissues are not negligible. In particular, wound healing patches should present features similar to those of human tissues, at the same time providing integrity and stability to offer a sufficient protective capability towards the external environment [43,44,45]. In this regard, electrospun membranes are characterized by a unique mechanical behaviour, which is related to their nanofibrous structure and can be easily modulated by changing the nanofiber size and spatial organization [11,46,47].

**Figure 4:**
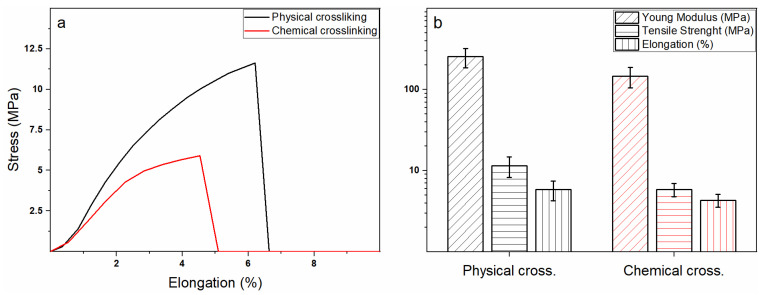
(**a**) Stress-deformation curves obtained from uniaxial tensile test for physically (black squares) and chemically crosslinked (red circles) electrospun mats. (**b**) Summary of the calculated mechanical properties (i.e., Young modulus E, elongation at break ε_b_, and tensile strength σ_b_).

Stress-deformation curves obtained for both physically and chemically crosslinked mats via uniaxial tensile test are reported in Figure 4a. Mechanical properties values (i.e., Young modulus E, elongation at break ε_b_, and tensile strength σ_b_) are summarized in Figure 4b.

Unexpectedly, the physical crosslinking led to more mechanically performing mats despite its weaker crosslinking efficiency. Specifically, the Young modulus and tensile strength were almost twice those of the chemically crosslinked membranes, whereas a slighter difference was observed for the elongation at break. However, rather than directly to the mechanism involved in the chitosan reticulation, the obtained disparities are most likely ascribable to the sample morphology. Indeed, the greater nanofiber homogeneity achieved via physical crosslinking led to the development of a much more ordered three-dimensional macroscopic structure able to provide for the superior mechanical performances. Conversely, the coalescence of the nanofibers occurring during the chemical crosslinking induces the establishment of a defect-rich network characterized by an inferior response [48,49].

Another key factor concerning wound healing patches is represented by their water-related behaviour [50,51]. As a matter of fact, such systems must be able to absorb a great quantity of exudate to keep the tissue dry (i.e., having high hydrophilicity) and allow a sufficient gas and vapour permeability (i.e., having a high porosity), at the same time being able to be stored over a long time (i.e., having a low moisture content). With this in mind, it is not surprising that electrospun membranes represent a promising class of materials for the fabrication of wound healing patches owing to their high surface area and porosity, which can easily meet the abovementioned requirements. However, the type of polymer, the fiber spatial organization, as well as post-production treatments (i.e., crosslinking, sterilization procedures, etc.) may influence the mat water-related behaviour and should be consequently taken into account.

The water contact angle, the water vapour permeability, and the moisture content values of both physically and chemically crosslinked chitosan-based mats are reported in Table 1.

First of all, a marked discrepancy between the hydrophilicity of the samples can be observed with the physically crosslinked mats showing a much lower WCA with respect to the chemically crosslinked ones. Such a result is related to both the crosslinking mechanism and nanofiber morphology. Chitosan, owing to the presence of residual acetyl groups, is characterized by a poor water solubility that is even lowered after the crosslinking approach. Therefore, the stronger the links between the macromolecular chains (i.e., chemical crosslinking), the smaller is the capability of chitosan to interact with water (i.e., greater hydrophobicity). Additionally, the thinner and defect-free nanofibers achieved via physical crosslinking present a higher surface area, thereby endowing the mats with a higher porosity, which, in turn, leads to capillarity effects that can promote the water-material compatibility. Further evidence of this phenomenon can be derived from the water vapour permeability value, which is nearly an order of magnitude smaller for the chemically crosslinked mats. In this regard, the presence of a great number of interconnected pores and the marked hydrophilicity of mats obtained via physical crosslinking seem to provide a much higher water vapour permeability. Finally, both samples presented low moisture content, hence being suitable to be stored over quite long periods without occurring in degradation issues and/or physical-chemical modifications.

Considering the above-discussed results, the physically crosslinked chitosan-based mats seem to be much more promising compared to the chemically crosslinked ones for the development of potential wound healing patches. Indeed, the achieved mechanical properties indicate that such systems could be able to endure considerable stresses and deformations before showing mechanical failure, thereby being suitable to provide a highly stable environment to foster cell viability in different types of wounds (e.g., surgical, traumatic, chronic, etc.). In addition, the marked hydrophilicity coupled with the great water vapour permeability may promote the exudate removal and gas exchange, thereby maintaining the tissues dried at the same time providing appropriate breathability, which are both important factors in the regeneration process.

### 3.3. Biological Response

**Figure 5:**
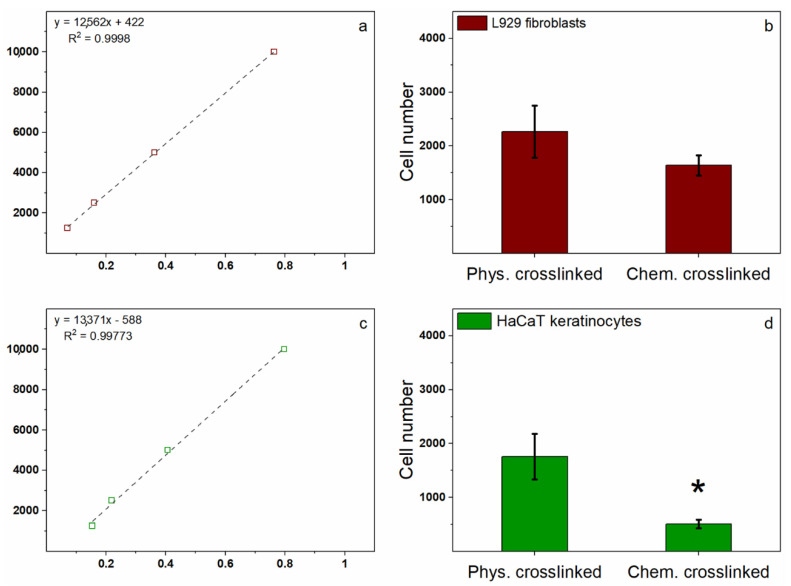

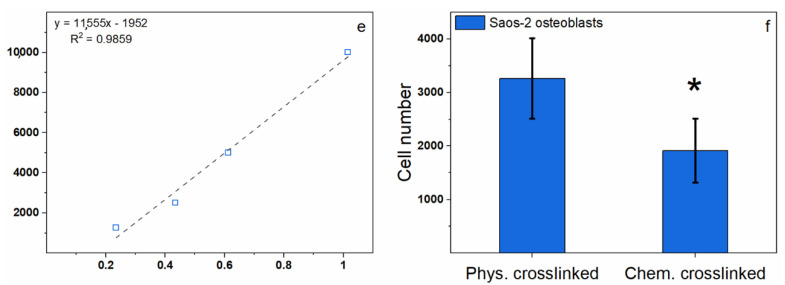
Cell adhesion evaluated by MTT test of attached cells after 16 h incubation. (**a**) L929 murine fibroblasts linear regression graph obtained by seeding doubling numbers of cells from 1250 to 10,000. The graph shows the trend line equation and the R2 data correlation. (**b**) L929 cell adhesion to the physically (Phys-cross) and chemically (Chem-cross) crosslinked membranes. Results are expressed as the number of adhered cells to each type of membrane obtained by interpolation to the standard regression curve shown in (**a**). They are the mean ± S.D. of 3 experiments performed in triplicate. (**c**) HaCat human keratinocytes linear regression graph obtained as in (**a**). (**d**) HaCaT cell adhesion obtained as in (**b**). Asterisk indicates significance in the *T*-test (* *p* < 0.05). (**e**) Saos-2 human osteoblasts linear regression graph obtained as in (**a**). (**f**)Saos-2 cell adhesion obtained as in (**b**). The asterisk indicates significance in the *T*-test (* *p* < 0.05).

**Figure 6:**
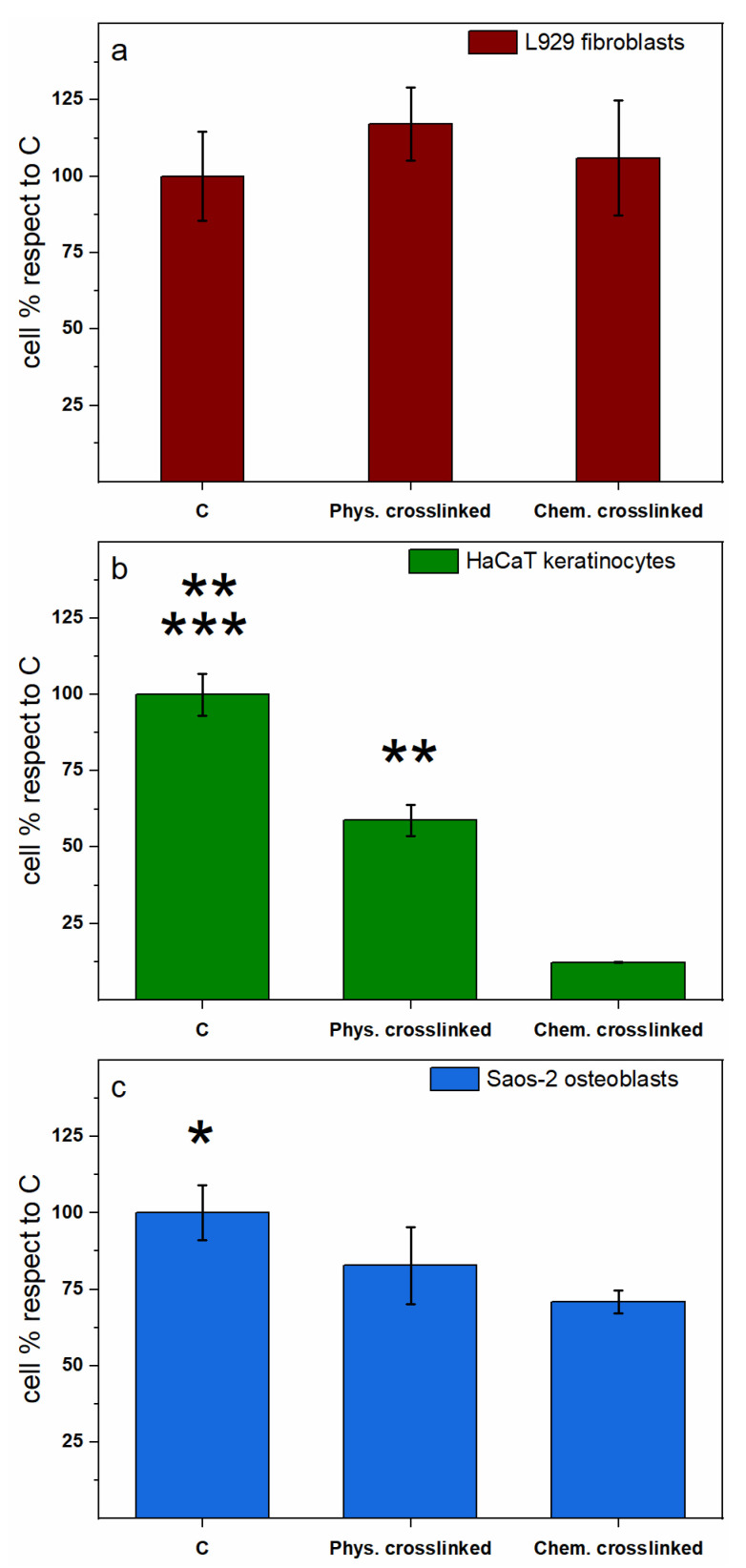
Cell toxicity, measured by MTT test, of conditioned culture media obtained by soaking the membranes for 24 h in sterile conditions at 37 °C and then adding the media diluted 1:1 to L929 murine fibroblasts (**a**), HaCaT human keratinocytes (**b**) or Saos-2 human osteoblasts (**c**) for further 24 h. Results are expressed as percentages respect to control, untreated cells and are the mean ± S.D. of 3 experiments performed in quadruplicate. The asterisk indicates significance in *T*-test (panel **b**: ** *p* < 0.0005 C vs. Phys-cross and Phys-cross vs. Chem-cross, *** *p* < 0.0001 C vs. Chem-cross, panel **c**: * *p* < 0.005 C vs. Chem-cross).

Chitosan is a well-known biocompatible material [28] even if the procedures usually performed to obtain 3D-dimensional scaffolds may affect the propensity of cells to adhere or grow on top of it due to the chemical modifications with respect to the original structure [34,35]. As a consequence, in this study, the two differently crosslinked chitosan membranes were tested for their biocompatibility by both measuring cell adhesion (Figure 5) and possible toxicity related to the release of chemicals that may be entrapped within the membranes and deriving from the reticulation procedures with negative effects on cell survival (Figure 6). Specifically, two skin cell lines, L929 fibroblasts and HaCaT keratinocytes, and an osteoblastic cell line, Saos-2, were used to assess the abovementioned parameters. Cell adhesion was measured by the MTT test after 16 h of incubation of 10000 cells per well onto the membranes in 96-well plates. To obtain the number of cells attached to the membranes, a parallel experiment was performed to derive a linear regression graph and the related trend line equation by seeding a standard curve of cells. For each cell type, the linear regression graph allowed us to interpolate the experimental values to obtain the total number of attached cells through the trend line equation (Figure 5a for fibroblasts, Figure 5c for keratinocytes, and Figure 5e for osteoblasts, respectively).

All cell types showed a clear preference for the physically crosslinked membranes with respect to the chemical ones. More in detail, the osteoblast cell line, Saos-2 (Figure 5f), showed the highest number of attached cells to the physically crosslinked membranes (32% respect to the number of seeded cells 16 h before) and this number was significantly higher than the 19% measured for the chemically crosslinked membranes (*p* < 0.05). The L929 fibroblasts (Figure 5b) showed a 22% of adhesion to the physically crosslinked membranes and, although not significant, this percentage was higher than the 16% adhesion measured on the chemically crosslinked membranes. Finally, the HaCaT keratinocytes (Figure 5d) showed the lowest values of cell adhesion on the chitosan membranes, with an 18% of cell adhesion obtained on the physically crosslinked membranes and a very poor 5% of adhesion to the chemically crosslinked membranes (*p* < 0.05). This latter result might be related to the fact that these cells preferentially adhere to fibrin or collagen substrates of protein origin. Such molecules, indeed, mimic more closely the basal membrane to which keratinocytes naturally adhere at the interface between epidermis and dermis [52]; therefore, a lower performance is expected from non-protein substrates such as chitosan or alginate. Overall, these data indicate that the bone cell line has a slightly superior preference than the skin cell lines for the chitosan membranes and that the physically crosslinked membranes are always preferred by all cell types compared to the chemical ones. The second important parameter evaluated was the cytotoxicity of chemicals eventually leaked from the membranes in the cell culture media that could affect both adhesion and proliferation of cells on the membranes. Thereby, the three cell lines were incubated for 24 h in presence or absence of the conditioned media obtained after a 24 h soaking of the two different chitosan membranes and cell viability measured by the MTT test (Figure 6).

Remarkably, a different degree of toxicity in the various cell lines was observed in the presence of the conditioned media from the physically and chemically crosslinked membranes compared to control untreated cells. In particular, two cell lines out of three—namely, HaCaT keratinocytes and Saos-2 osteoblasts—were significantly affected by the conditioned media from the 24 h soaking of the chemically crosslinked membranes, with a 88% and 30% cell mortality (Figure 6b and Figure 6c, respectively) compared to control cells (*p* < 0.0001 and *p* < 0.005, respectively). Conversely, only one cell line—namely, HaCaT keratinocytes reported in Figure 6b—was significantly affected by the conditioned medium from the soaking of the physically crosslinked membranes, with a cell mortality of 42% compared to control cells (*p* < 0.0005). In contrast, no toxicity was observed in the L929 fibroblasts (Figure 6a) either in presence of the conditioned media from the physically either from the chemically crosslinked membranes, which indicates them to be the most resilient cell type. In general, these data seem to indicate that the chemically crosslinked membranes release some chemicals in the culture media towards which cells show a different, and sometimes opposite, sensitivity, with fibroblasts growing completely unaffected, osteoblasts slightly affected, and keratinocytes that seem not able to survive in the presence of the released chemical (Figure 6b). Since the chemically crosslinked membranes use EGDE to reticulate chitosan, residues of this molecule could likewise leak from the scaffolds causing cell death. This toxicity could also be one of the reasons why keratinocytes show the lowest adhesion capacity with respect to the other cell types (Figure 5d), other than being the chitosan membranes a non-protein substrate. Conversely, the conditioned media from the physically crosslinked membranes show only some toxicity on the HaCaT keratinocytes (Figure 6b), although anyway a 18% cell adhesion on these membranes is measured (Figure 5d), not far from the percentage measured for fibroblasts (22%, Figure 5b) that show no toxicity at all in the same conditions. Overall, it is possible to conclude that the physically crosslinked membranes show significant better performances in terms of cell adhesion and low toxicity indicating these as the most suitable materials for the production of wound healing patches.

### 3.4. Drug Delivery Properties

**Figure 7:**
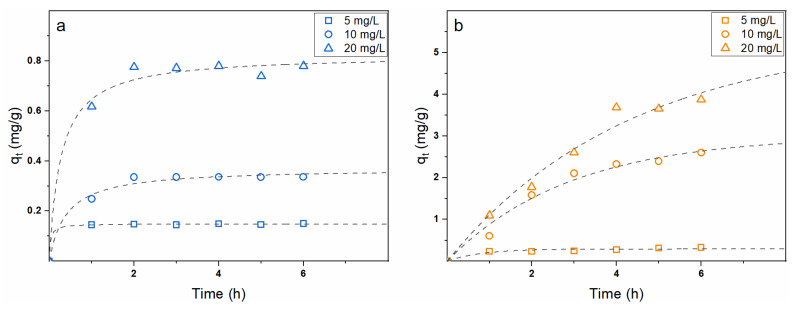

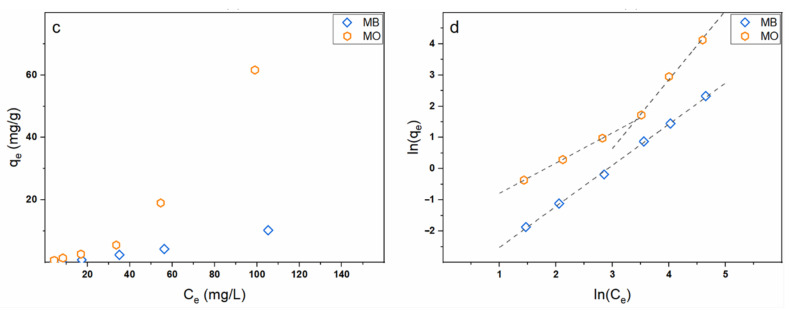
Adsorption kinetics of physically crosslinked chitosan membranes in (**a**) MB and (**b**) MO solutions with different dye concentration (i.e., 5, 10, and 20 mg/L). Data fitting with the pseudo-first-order kinetic model is reported in dashed lines. (**c**) Adsorption isotherm data of physically crosslinked chitosan membranes and (**d**) their fitting with the Freundlich model. q_t_, q_e_, and C_e_ represent the adsorption capacity at a certain time t, the adsorption capacity at equilibrium, and the dye solution equilibrium concentration, respectively.

To date, most of the commercially available wound healing patches have the unique role of promoting tissue regeneration and/or by foster cell adhesion and proliferation [43]. In this regard, the main factors affecting their efficiency are the employed materials and their three-dimensional structure. Nevertheless, the development of wound dressing systems endowed with controlled drug delivery capabilities represents an interesting but poorly successful research field [43,53,54]. Consequently, in the present work, owing to their promising physical-chemical features and good biological response, the proposed physically crosslinked chitosan-based mats were explored as DDSs by their loading with MB and MO dyes, which were selected as positively and negatively charged drug models, respectively. First of all, the possibility to use a simple impregnation approach to upload the colourants within the mats was evaluated by using dye solutions at increasing concentration. Figure 7a,b report the adsorption kinetics for MB and MO, respectively. Independently on the colourant type, increasing both the solution concentration and contact time corresponded to increase the membrane adsorption capacity. However, significant differences were observed with respect to the investigated dye.

In detail, MB was scarcely adsorbed by the mats with the process being almost arrested after only 2 h for the studied experimental conditions. Conversely, MO was rapidly and much more efficiently entrapped within the electrospun membranes with the phenomenon evolving even after 6 h, especially for the highest dye concentration (i.e., 20 mg/L). To better understand the kinetics of adsorption, pseudo-first-order and pseudo-second-order models were employed to fit the experimental data [55,56]. Interestingly, it was possible to describe the adsorption of MB by means of the pseudo-second-order model, whereas only the pseudo-first-order model was successfully applied to the MO data. The model calculated parameters (i.e., equilibrium adsorption efficiency q_e_ and rate constants k_1_ and k_2_) are summarized in Table 2.

The results suggest that a different adsorption mechanism occurs for MB and MO dyes, which is in agreement with their different charge. Specifically, MB being positively charged and taking into account the low q_e_ values, it can be supposed that strong repulsive interactions arise between chitosan chains and the colorant molecules. Therefore, the adsorption is most likely driven by diffusive phenomena but remains highly hindered due to the electrostatic repulsions. In this sense, since oxygen-containing groups were proved to be frequently involved in the adsorbate-adsorbent mechanisms, the hydroxyl substituents of chitosan are likewise supposed to provide functional sites for the uptake through hydrogen bonding [57]. Conversely, owing to their opposite charge, chitosan and MO molecules can attractively interact, thus enhancing the adsorption efficiency, as proved by the achieved higher q_e_ values. Nevertheless, since the adsorption process continues over a certain period of time, it is most likely that both electrostatic and diffusive phenomena play here an important role.

In this sense, further confirmation was obtained by studying the isotherms of adsorption for both MB and MO dyes with the related results shown in Figure 7c. Clearly, much higher adsorption efficiencies were obtained for MO, especially at high dye concentration, thereby demonstrating the greater affinity between the negatively charged colourant and chitosan compared to MB. The experimental data were fitted with both Langmuir [58,59] and Freundlich [59,60] models, with the first completely failing in describing the adsorption process, hence confirming the effect played by both electrostatic and diffusive phenomena. As such, the linear data fitting according to the Freundlich model is shown in Figure 7d and the calculated parameters are summarized in Table 2. Specifically, Freundlich model relies on the assumption that the adsorption phenomenon occurs on a heterogeneous surface owing to the presence of numerous binding sites, which, in turn, leads to the formation of several adsorbate multilayers. The greatest difference in the isotherm adsorption process for the two explored dyes is the presence of two distinct regions for MO, whereas a single one is observed for MB. Such finding is likewise ascribable to the involved adsorption mechanism. As a matter of fact, MB dye is being entrapped within the mats only via a diffusion mechanism with the occurring electrostatic repulsions reducing the process efficiency and a unique adsorption region is observed in the whole investigated concentration range. On the contrary, both attractive electrostatic interactions and diffuse phenomena are involved in the adsorption of MO with one being predominant on the other depending on the dye concentration. In this regard, more information regarding the adsorption process can be derived from the slope of Freundlich linear fitting (i.e., 1/n), which is a parameter indicating of the isotherm type and providing important insights on the intensity of the mechanism. 1/n values lower than 1 are commonly found in systems where the adsorption sites can be easily saturated (i.e., the adsorption process is mainly driven by electrostatic interactions). Conversely, 1/n values higher than 1 are indicative of S-isotherms and usually occurs when polar molecules compete with water for the adsorption sites (i.e., the adsorption process is mainly driven by diffusive mechanisms). Therefore, the existence of the two distinct regions for MO is ascribable to the predominance of electrostatic adsorption at a low dye concentration, whereas diffusive adsorption assumes a major role once the electrostatic binding sites are saturated (i.e., high dye concentration).

**Figure 8:**
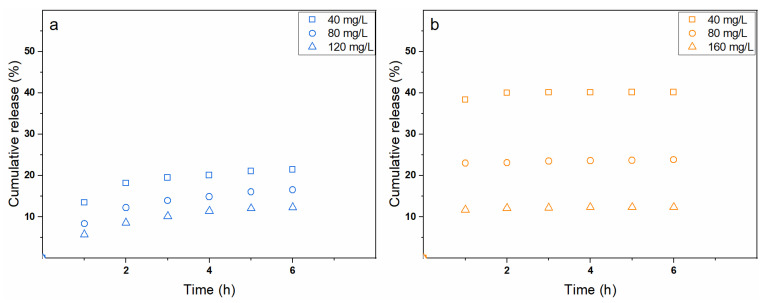
Cumulative release from the physically crosslinked chitosan membranes in PBS after their loading in (**a**) MB or (**b**) MO solutions with different dye concentration (i.e., 40, 80, and 160 mg/L).

Independently on the phenomena driving the dye upload, a much more interesting aspect is related to the membrane release capabilities that are of topical importance in the development of controlled drug delivery systems. Figure 8a,b show the dye cumulative release in simulated physiological conditions (i.e., phosphate buffer saline at pH = 7.4 and T = 37 °C) for MB and MO, respectively, depending on the concentration of the loading solutions.

Calculated data are reported in Table 2. Remarkably, MB and MO dyes showed different release kinetics. Indeed, MO was rapidly released from the mats in the first hour, whereas MB displayed a sustained release over a time period of 6 h. Such differences are likewise related to the different charge of the dyes. Specifically, the negative ions in PBS can screen the positive charges of chitosan, hence forcing the almost immediate release of the electrostatically adsorbed dye, whereas a consistent quantity of the diffusively adsorbed colourant remains within the nanofibers as proven by the relatively low R% values. On the contrary, a small fraction of MB dye is slowly released from the mats, which is consistent with its adsorption mechanism. However, it should be noted that in both cases, the total amount of released colourants was a function of the uploading concentration, thereby suggesting the possibility to accurately modulate the release phenomena.

In addition, future studies will be required, the achieved results represent a promising starting point in the development of simple, cheap, and efficient drug delivery systems with modulable release kinetics. Indeed, despite negatively charged molecules presenting a higher affinity for chitosan-based nanofibers and positively charged ones showing a better-controlled release, depending on both the drug nature and loading concentration, it could be possible to obtain DDSs able to provide a burst or a slow-release to satisfy the specific requirements.

## 4. Conclusions

In the present work, electrospun chitosan-based nanofibers were prepared and subsequently subjected to a physical or chemical crosslinking treatment by using phosphate ions or ethylene glycol diglycidyl ether, respectively. Remarkably, physically crosslinked membranes were proved to show a broad number of advantages with respect to the chemically crosslinked ones. In particular, the use of phosphate ions led to much thinner and homogenous nanofibers (i.e., average size of 190 nm), which, in turn, provided a greater global porosity and an enhanced mechanical response, as well as more marked hydrophilicity and water vapour permeability. Above all, significant differences were depicted between the mats in terms of cell adhesion and cytotoxicity. In particular, the nanofibers treated with phosphate ions showed no toxicity towards the different tested cell lines along with the capability to strongly promote their adhesion and proliferation, especially for osteoblasts. Conversely, the chemically crosslinked mats were found to be toxic for keratinocyte and osteoblast cell lines, thereby being able to marginally foster cell viability most likely due to the release of some residual chemicals. Owing to the aforementioned findings, the physically crosslinked mats were then loaded with two dyes, which were used as model drug molecules, and their delivery properties were intensively evaluated. Interestingly, by simply changing the charge of the dyes, the mats were proved able to show a burst or a slow sustained release, hence being suitable for different routes of administration. In conclusion, the proposed physically crosslinked chitosan-based mats represent a potential class of innovative, cheap, and highly efficient materials to be used as advanced wound healing patches with tuneable drug delivery properties. However, it is noteworthy that further in vitro and in vivo experiments are required to open the way to their effective use in biomedical and pharmaceutical products. Among others, some critical points that must be soon addressed include the improvement of the fabrication procedure (i.e., higher production rate and reproducibility), a better understanding of the product biological response (i.e., cell migration and differentiation) and drug delivery properties, as well as the evaluation of the scaffold degradation kinetics in physiological conditions.

## Figures and Tables

**Table 1:** Summary of the water-related properties (i.e., water contact angle WCA, water vapour permeability WVP, and moisture content MC) evaluated for chitosan-based membranes.

Crosslinking Type	WCA (°)	WVP (g/s·m·Pa)	MC (%)
Physical	56 ± 5	9.3 **·** 10^−12^	11 ± 2
Chemical	71 ± 2	2.1 **·** 10^−12^	10 ± 1

**Table 2:** Summary of the calculated parameters for the adsorption kinetics, adsorption isotherms, and cumulative release.

Dye	Adsorption Kinetics			Freundlich		Cumulative Release	
	C (mg/L)	Pseudo-First Order	Pseudo-Second Order				
		q_e_ (mg/g)	q_e_ (mg/g)	k_F_ ((mg·(L/mg)^1/n^)/g)	1/n	C (mg/L)	R%
MB	5 10 20	---	0.15 0.37 0.82	2.0**·**10^−2^	1.31	40 80 160	12 17 21
MO	5 10 20	0.29 3.00 5.58	---	17.0**·**10^−2^ 0.2**·**10^−2^	0.97 2.21	40 80 160	12 24 40

## Data Availability

Data will be made available at request.
